# Welcome to our new Section Editors

**DOI:** 10.1007/s15010-023-02026-7

**Published:** 2023-04-17

**Authors:** Johannes R. Bogner

**Affiliations:** Sektion Klinische Infektiologie, Med. Klinik und Poliklinik IV, Klinikum der Universität München, Pettenkoferstr. 8a, 80336 Munich, Germany

INFECTION was founded in 1973 and will celebrate its 50th birthday this year. It was founded upon the initiative of Prof. Walter Marget, who was a pediatrician and microbiologist [1]. The aim of the journal has been and remains till today to serve the scientific communication with respect to all aspects of clinical infectious diseases embracing all age groups from newborns up to geriatric aspects of infectious diseases.

The international claim and the international orientation of a modern journal are shown by the fact that manuscripts, submissions, and publications from all continents and from all fields of infectious medicine occur. The COVID-19 pandemic has once again made it clear how important vaccination is [2–5]. So what could be better than welcoming a new Section Editor who is pediatric trained and covers aspects of vaccine preventable pediatric infectious diseases in addition to specialization in vaccinology: we are grateful and proud that Prof. Helen Marshall from Adelaide, Australia has agreed to follow in the footsteps of Walter Marget and David Nadal as the new Section Editor for Pediatric Infectious Diseases.

Prof. Marshall undertook her pediatric training at the Women's and Children's Hospital in Adelaide, where she saw the impact of serious infections from diseases without vaccines available. Marshall focused on research in vaccinology, public health, and infectious diseases [6–9]. She is a Professor of Vaccinology in the Adelaide Medical School and Deputy Director, Clinical and Translational Research for the Robinson Research Institute at the University of Adelaide. She is also a Consultant in Vaccinology and Medial Director for Vaccinology and Immunology Research Trials Unit at the Women's and Children's Hospital and the inaugural Clinical Research Director. She was awarded with numerous fellowships, grants and prizes and she serves as an advisor to the World Health Organization. She has published over 250 peer-reviewed papers and has been awarded numerous research grants [10]. In October 2021, Marshall was named South Australian of the Year for her work in public health and infectious diseases, making her the state's nominee for Australian of the Year [6]. She was made a Member of the Order of Australia in the 2022 Australia Day Honours [11].

The COVID-19 pandemic has also once more shown that fungal diseases are important killers in infectious diseases. COVID-19-associated aspergillosis (CAPA) and also with COVID-19-associated mucormycosis (CAM) have been extensively covered by Infection [12, 13]. These and other systemic fungal diseases will remain to constitute part of the scientific content of our journal. We are happy to announce that Prof. Rita Oladele from Nigeria has agreed to join the journal to be the editor in the section of clinical manifestations, epidemiology and therapy of mycoses along with Prof. Birgit Willinger from Vienna, who joined INFECTION in 2019 and who represents the laboratory part of fungal research, diagnostics and pathophysiology.

Prof. Oladele is an Associate Professor of Medical Microbiology and Parasitology at the University of Lagos in Nigeria. One of her latest projects is one that reaches out to patients far away from medical centers: telemedicine and education in mycology. One of her concerns is that invasive mycoses may be overseen by physicians who are not well trained in infectious diseases including mycoses [14]. Another topic of Prof. Oladele is histoplasmosis and its prevalence in Africa and all over the world [15]. She has authored 62 PubMed-listed publications and she is a member of international boards editing guidelines and recommendations regarding fungal diseases [16, 17].

With both the new Editors, INFECTION steers its way through a global world with better access to scientific content. More countries and continents are represented at our journal and we take another step to reach gender equality in our boards. I look forward to our collaboration in the team of editors supported by the editorial board.

We would also like to thank all our readers and scientists who have submitted and will continue to submit their excellent publications to INFECTION. Thank you for your loyalty.

January 2023, Johannes Bogner

Editor-in-Chief of INFECTION
